# Hysterectomy for cervical intraepithelial neoplasia: A retrospective observational multi‐institutional study

**DOI:** 10.1002/ijgo.14233

**Published:** 2022-05-12

**Authors:** Andrea Ciavattini, Jacopo Di Giuseppe, Chiara Marconi, Luca Giannella, Giovanni Delli Carpini, Michela Paolucci, Mariasole Fichera, Rosa Pasqualina De Vincenzo, Giovanni Scambia, Maria Teresa Evangelista, Giorgio Bogani, Francesca Bertolina, Francesco Raspagliesi, Barbara Gardella, Arsenio Spinillo, Mattia Dominoni, Ermelinda Monti, Carlo Antonio Liverani, Paolo Vercellini, Maria Iorio, Domenico Vitobello, Rosalba Portuesi, Gianluigi Bresciani, Massimo Origoni, Francesco Cantatore, Antonio Maurizio Pellegri, Lorenzo Moriconi, Matteo Serri, Andrea Chiari, Francesco Sopracordevole, Maggiorino Barbero, Fabio Parazzini, the Italian Society of Colposcopy and Cervico‐Vaginal Pathology (SICPCV)

**Affiliations:** ^1^ Obstetrics and Gynecologic Section, Department of Odontostomatologic and Specialized Clinical Sciences Università Politecnica delle Marche Ancona Italy; ^2^ Gynecologic Oncology Unit, Department of Woman and Child Health and Public Health Fondazione Policlinico Universitario A. Gemelli, IRCCS Rome Italy; ^3^ Dipartimento di Scienze della Vita e Sanità Pubblica Università Cattolica del Sacro Cuore Rome Italy; ^4^ Gynecologic Oncology Unit Fondazione IRCCS Istituto Nazionale dei Tumori di Milano Milan Italy; ^5^ Department of Obstetrics and Gynecology Istituto di Ricovero e Cura a Carattere Scientifico (IRCCS) Foundation Policlinico San Matteo and University of Pavia Pavia Italy; ^6^ Gynecology Unit, Fondazione IRCCS Ca' Granda Ospedale Maggiore Policlinico Milan Italy; ^7^ IRCCS Humanitas Research Hospital, Rozzano Milan Italy; ^8^ Vita Salute San Raffaele University School of Medicine ‐ Department of Gynecology & Obstetrics Milan Italy; ^9^ Obstetrics and Gynecologic Unit, A.S.U.R Benedetto del Tronto (AP) Italy; ^10^ Gynecological Oncology Unit Centro di Riferimento Oncologico di Aviano (CRO) IRCCS Aviano Italy; ^11^ Department of Obstetrics and Gynecology Asti Community Hospital Asti Italy; ^12^ Department of Clinical Sciences and Community Health Università degli Studi di Milano Facoltà di Medicina e Chirurgia Milan Italy

**Keywords:** cervical intraepithelial neoplasia, conization, human papillomavirus, hysterectomy, vaginal cancer, vaginal intraepithelial neoplasia

## Abstract

**Objective:**

To analyze the clinical management, the outcomes, and the trend in hysterectomy rates (HR) in patients who underwent this procedure for cervical intraepithelial neoplasia (CIN).

**Methods:**

Multicentric retrospective observational study conducted on 242 patients who underwent hysterectomy for CIN between 2010 and 2020 in nine Italian institutions. Hysterectomy for invasive or micro‐invasive neoplasia, sub‐total hysterectomy, or trachelectomy were excluded.

**Results:**

A significant increase in the trend of HR for CIN was recorded (*P =* 0.002, *r* = 0.81; C.I. 95%: 0.415–0.949); HR increased from 0.46% in the year 2010 to 3.32% in 2020. The mortality rate was 0.4%, and 5% had operative complications. On definitive histopathology examination, a CIN of any grade was recorded in 71.5% of cases, and an occult invasive cancer in 1.24%. No pathology or CIN1 was found in 26.8% of cases, suggesting over treatment. During follow‐up, a vaginal lesion was recorded in 5% of cases.

**Conclusion:**

A significant increase in the number of hysterectomies performed for CIN in the last 10 years was recorded. Hysterectomy for CIN can lead to complications, risk of the onset of vaginal lesions, and risk of overtreatment, and remains, in the first instance, an unacceptable treatment, to be proposed only after adequate counseling.

## Synopsis

The contents of this page will be used as part of issue TOC only. It will not bepublished as part of main article.Hysterectomy for CIN can lead to complications and risk of overtreatment, and remains, in the first instance, an unacceptable treatment.

## 1 INTRODUCTION

The primary objectives of the management of the patient with a histological high‐grade squamous intraepithelial lesion (HSIL) (cervical intraepithelial neoplasia, CIN 2–3) are to eliminate the cervical cancer precursors and to identify occult micro‐invasive or invasive carcinoma early on, thus reducing mortality from cervical cancer.^1^, ^2^, ^3^, ^4^ When treatment is planned, an excisional procedure (LEEP, cold knife cone, and laser cone biopsy) is preferred and must be carried out under colposcopic vision.^5^ In the last 20 years, a significant decrease in the length of cone excision was observed.^6^ This can be because of the gained awareness of the potential disadvantages of wide cone excision in terms of adverse obstetric outcomes in future pregnancies.^6^

Every year, in Italy, over 50 000 hysterectomies are performed: 75% for benign pathologies such as recurrent menorrhagia or uterine fibroids, less frequently for prolapse.^7^ In addition, many patients with cervical intraepithelial neoplasia (CIN) are still being treated with hysterectomy, despite it not being considered an acceptable treatment.^8^, ^9^, ^10^, ^11^, ^12^, ^13^

In 2019 recommendations of the Italian Society of Colposcopy and Cervical‐Vaginal Pathology (SICPCV),^14^ and in the Italian guidelines of 2002 and 2006,^14^ in the presence of cervical HSIL, hysterectomy is defined as an unacceptable treatment. This procedure is acceptable, after informed consent, only if it is not possible to carry out or to repeat a diagnostic excision or in case of an impossible adequate follow‐up. For example, when multiple treatments, technical difficulties in the surgical approach to the residual cervix, untreatable stenosis of the cervical canal, vaginal stenosis are encountered. In the absence of other indications, and regardless of the age and obstetric history of the woman, hysterectomy could be harmful because of surgical complications and for the limitations of subsequent follow‐up.

Some reports highlighted that the risk of vaginal cancer or vaginal intraepithelial neoplasia (VaIN) is significantly increased (5%–10%) in women who have had a hysterectomy for cervical cancer or CIN.^15^, ^16^, ^17^, ^18^, ^19^, ^20^, ^21^ A recent study,^21^ evaluating the outcome of a large cohort of Swedish patients undergoing hysterectomy for CIN, found an incidence rate of vaginal cancer of 51.3 per 100 000. The risk of vaginal cancer was increased after hysterectomy for women with benign cervical history as well (Incident Rates, IR 3.65). Compared to the general population (IR‐ratios 0.37) their risk was more than doubled. Very few other studies are available in the literature. Among these, a Dutch study^22^ pointed out that about 3% of CIN3 is still being treated with hysterectomy.

Data about trend of hysterectomy rate performed for CIN and about outcomes of patients with CIN undergoing this procedure are limited, and the compliance with national and international guidelines has been poorly assessed, making clear the importance of carrying out further studies.

The present study was aimed at analyzing the clinical management, the outcomes, and the trend in hysterectomy rates in patients who underwent this procedure for CIN between 2010 and 2020, across Italian referral centers. The study was conducted under the patronage of the SICPCV.

## 2 MATERIALS AND METHODS

This retrospective cross‐sectional multi‐institutional Italian study included women who consecutively underwent total hysterectomy for CIN between January 2010 and December 2020. The study design provides that the procedures and data of interest had already been performed or gained according to routine clinical practice before starting the study. All patients signed informed consent for data collection for scientific research. The Institutional Review Board (IRB) of the Azienda Ospedaliera Ospedali Riuniti di Ancona (Italy) approved the study (IRB#9–2021).

The nine centers taking part in the study were: Obstetrics and Gynecologic Section, Department of Odontostomatologic and Specialized Clinical Sciences, Università Politecnica delle Marche, Ancona, Italy; Gynecologic Oncology Unit, Department of Woman and Child Health and Public Health, Fondazione Policlinico Universitario A. Gemelli, IRCCS, Roma, Italy; Department of Gynecology & Obstetrics, Ospedale San Raffaele Scientific Institute, Vita‐Salute San Raffaele University School of Medicine, Milan; Gynecologic Oncology Unit, Fondazione IRCCS Istituto Nazionale dei Tumori di Milano, Milan, Italy; Department of Obstetrics and Gynecology, Istituto di Ricovero e Cura a Carattere Scientifico (IRCCS) Foundation Policlinico San Matteo and University of Pavia, Pavia, Italy; Department of Clinical, Surgical, Diagnostic, and Pediatric Sciences, University of Pavia, Pavia, Italy; Gynecology Unit, Fondazione IRCCS Ca′ Granda Ospedale Maggiore Policlinico, Milano, Italy; IRCCS Humanitas Research Hospital, Rozzano, Milan, Italy; Vita Salute San Raffaele University School of Medicine ‐ Department of Gynecology & Obstetrics, Milano, Italy; Obstetrics and Gynecologic Unit, A.S.U.R., Area Vasta 5, S. Benedetto del Tronto (AP), Italy.

All patients included in the study had to have a time interval between the last diagnosis of CIN (conization or biopsy) and hysterectomy of less than 12 months, to reduce the bias related to the risk of progression of CIN in the interval between the two procedures.

Patients undergoing hysterectomy for invasive or micro‐invasive neoplasia of the lower genital tract, or undergoing sub‐total hysterectomy, or trachelectomy were excluded.

The primary outcome measure was the proportion between the number of hysterectomies performed for CIN and those performed for the other benign gynecological pathologies, excluding CIN. Secondary outcomes were the mortality and morbidity rates and rate of histopathology upgrading or downgrading at post‐operative examination. Data from the DRG statistics were extracted for the period 2010–2020. The ICD‐10 codes relevant for hysterectomy were selected and the indication for surgery was identified. All data were collected from medical records.

For each patient the following data were collected: age, menopausal status, ethnicity, educational qualification, presence of comorbidities, BMI, parity, participation in organized screening, smoking habit, previous abdominal surgery, any associated pathology both gynecological and extra gynecological (breast cancer–Breast Cancer gene [BRCA] mutation if known; urinary incontinence; other neoplastic pathology), family history of gynecological and/or mammary neoplastic pathology; the number and type of cervical treatments performed with the relative histological examinations and the human papillomavirus (HPV) status (and HPV subtype), the pre‐hysterectomy histological examination from biopsy and/or cervical excision/or conization, the indication for hysterectomy (“CIN alone” or “other indication besides CIN”), the definitive histological examination of the hysterectomy with finding of up‐grading compared to the pre‐operative histological examination, the possible diagnosis of occult adenocarcinoma in situ (AIS) lesions (not diagnosed in the pre‐operative examinations) or of lesions of the uterine body (such as endometrial cancer). The surgical technique performed, its duration, the occurrence of intra or post‐operative complications, the need for blood transfusions have been evaluated. Months of follow‐up and incidence of VaIN or vaginal cancer were also recorded.

### 2.1 Statistical analysis

All continuous variables were tested for normality with the D'Agostino‐Pearson test; normally distributed variables were expressed as mean ± SD, while skewed variables were reported as median and range. The trends of the variables included, as a function of the year of the procedure, were analyzed with Pearson correlation coefficient or with Spearman rank correlation method when distribution was normal or not normal, respectively.

Qualitative variables were expressed as proportions and were compared with Chi‐square or Fisher's exact test, as appropriate. A bivariate analysis was performed to identify factors that were significantly associated with the incidence of occult micro‐invasive and invasive cancer in patients hysterectomized for CIN. A stepwise multivariate linear regression analysis was then performed, including factors that were significantly associated with the incidence of micro‐invasive cancer in patients hysterectomized for CIN. A *P*‐value of less than 0.05 was considered being statistically significant.

The sample size calculation was based on the expected HR. According to previous retrospective data (23), the percentage of women who underwent total hysterectomy mainly for CIN was about 3%. With a Type I error ‐ alpha of 0.05, a Type II error ‐ beta of 0.2 (power = 80%), and a null hypothesis value of 2.5%, the total sample size will need 8085 women (expecting a percentage of women who underwent hysterectomy mainly for CIN in the last 10 years of 3%).

All statistical analyses were performed using MedCalc Statistical Software (MedCalc® Statistical Software version 19.5.3; MedCalc Software Ltd, Ostend, Belgium; https://www.medcalc.org; 2020). A *P*‐value <0.05 was considered statistically significant.

## 3 RESULTS

**FIGURE 1:**
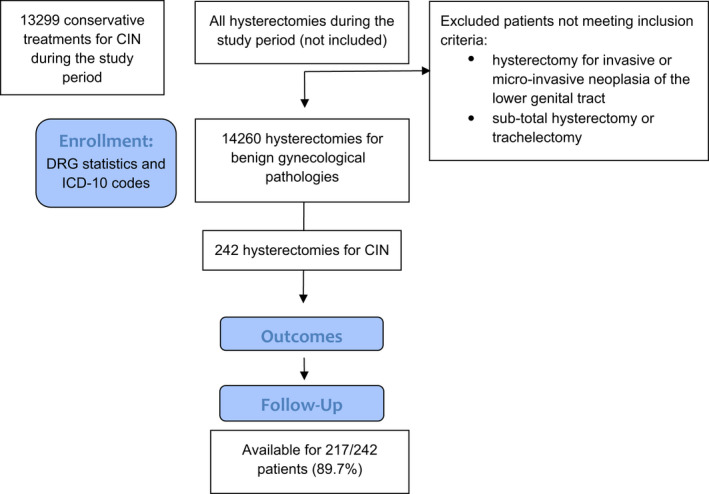
Study flow chart

During the study period, 14 260 hysterectomies were consecutively performed for benign gynecological pathologies, and 242 hysterectomies (1.7%) had CIN as the main indication and represented the study group (Figure 1).

**FIGURE 2:**
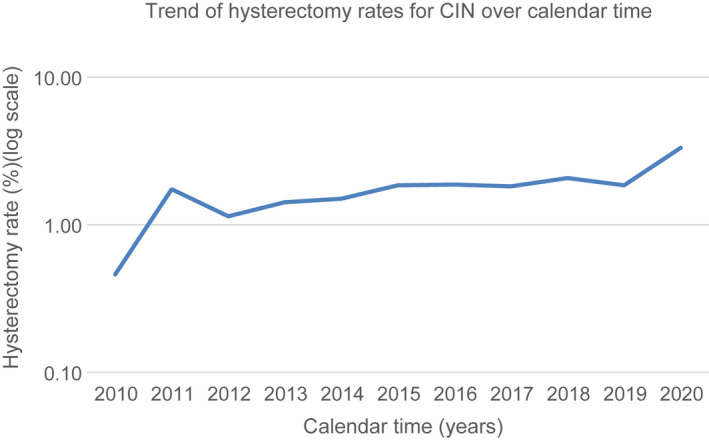
The trend of hysterectomies performed for CIN in the period 2010–2020 (total number of hysterectomies: 14 260) (expressed as rates)

The proportion between the number of hysterectomies performed for CIN and those performed for the other benign gynecological pathologies excluding CIN varied from 0.46% in the year 2010 to 3.32% in 2020. A significant increase in the trend of hysterectomy rate (HR) for CIN was recorded (*P =* 0.002, *r* = 0.81; C.I. 95%: 0.415–0.949) (Figure 2).

Similarly, during the study period, 13 299 conservative treatments for CIN were consecutively performed, and the proportion between hysterectomy and excisional treatment for CIN increased from 0.79% (2010) to 3. 13% (2020) (mean 1.93, SD 0.79), with a significant upward trend (*P* ≤ 0.001, *r* = 0.87; C.I. 95%:0.5698–0.9663) (Table 1).

**TABLE 1:** The proportion between the number of women with CIN treated with hysterectomy and with cervical excision according to years of the procedure

Year	Cervical excision for CIN (n°)	Hysterectomies for CIN (n°)	Proportion (%)
2010	758	6	0.79
2011	1531	23	1.50
2012	1221	14	1.14
2013	1491	19	1.27
2014	1770	22	1.24
2015	1255	25	1.99
2016	872	26	2.98
2017	932	22	2.36
2018	1045	27	2.58
2019	1162	26	2.23
2020	1020	32	3.13

The characteristics of the study group are shown in Table 2. The median age was 50 years (range: 30–79 years) and the median BMI was 24 kg/m^2^ (range: 13.3–44 kg/m^2^). 65% of patients adhered to cervical cancer screening; 72% had one or more pregnancies and no woman had a desire to preserve fertility. Family history for gynecological or breast cancer was present in 11% of patients. All patients had almost a previous conization, and 24% of them had two or more conizations. Further conservative treatment was described as “technically not feasible” before proceeding with hysterectomy only in nine cases (3.7%).

**TABLE 2:** Baseline characteristics of the study population

Characteristics	Variables	Frequency (n°)	Percentage (%)
Median age (years) (range)	50 (30–79)		
Median BMI (kg/m^2^) (range)	24 (13.3–44)		
Education	Primary school	26	10.74
	Secondary School	59	24.38
	Degree	24	9.92
	Unknown	133	54.96
Ethnicity	Caucasian	230	95.04
	Asian	5	2.07
	Middle Eastern	2	0.82
	South American	5	2.07
Menopause	No	124	51.25
	Yes	117	48.35
Family history	Negative	215	88.84
	Breast cancer	14	5.79
	Gynecologic cancer	13	5.37
Comorbidity (almost one)	Cardiovascular disease	46	19
	Diabetes	5	2.07
	Kidney disease	7	2.89
	Liver disease	1	0.41
	Other neoplasia	9	3.72
	Breast cancer	12	4.96
	Urge incontinence	2	0.83
	Immunodeficiency	5	2.07
	None	168	69.42
Smoking status	No	177	73.14
	Yes	63	26.03
	Unknown	2	0.83
Previous abdominal surgery	No	120	49.59
	Yes	122	50.41
Parity	Nulliparous	67	27.68
	1	60	24.79
	2	89	36.78
	3	24	9.92
	4	2	0.83
Cervical cancer screening adherence	No	62	25.62
	Yes	157	64.88
	Unknown	23	9.50
CIN as main indication for surgery	No	0	0
	Yes	242	100
Additional indication for surgery to CIN	Fibromatosis/adenomyosis	17	7.02
	Prolapse	3	1.24
	Menometrorrhagia	2	0.83
	Other	6	2.48
	Ovarian cyst	3	1.24
	hyperplasia – endometrial polyposis	5	2.07
	HG‐VaIN	2	0.83
	None	204	84.29

**FIGURE 3:**
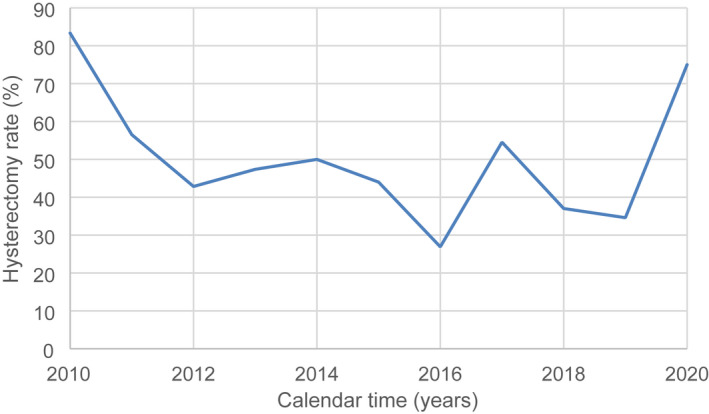
The trend of patients in menopause underwent hysterectomies for CIN in the period 2010–2020 (total number of hysterectomies for CIN: 242) (expressed as rates)

No variation in the trend of median age (*P =* 0.499, *r* = −0.229; C.I. 95%: −0.729–0.430) emerged during the study period. The rate of menopausal patients who underwent hysterectomy for CIN showed a slight decrease (*P =* 0.41, *r* = −0.27; C.I. 95%: −0.752‐0.385) (Figure 3) except for the last 2 years, when a significant increase was recorded (61.54% vs. 38.46%, *P =* 0.01, C.I. 95%: 4.843–39.779). Similarly, no other change in the characteristics of the patients was observed during the study period, including the surgical technique used to perform the hysterectomy.

The pre‐hysterectomy cervical cytological and histopathological findings are shown in Table 3. 84.3% of patients had high‐grade cytology (≥ASCH), and 67.4% of them had positive HR‐HPV test. The histopathological diagnosis was got with conization in 192 patients (79.3%), and in 50 patients (20.7%) with cervical biopsy performed during follow‐up for CIN. A grading ≥ CIN2 was observed in 90.1% of patients. In the remaining 17 patients (9.9%) who had a grading ≤ CIN1, 53.3% was in follow‐up for CIN ≥2, and 46.7% was for persistent CIN1; 40% of them had two or more previous conizations.

**TABLE 3:** Preoperative cytological, histopathological, and cervical HPV‐related history of the study population

Characteristics	Variables	Frequency (n°)	Percentage (%)
Cervical cytology	ASC‐US	7	2.89
	LSIL	12	4.96
	HSIL	192	79.34
	AGC‐NOS	5	2.07
	ASC‐H	8	3.30
	Squamous cell carcinoma	4	1.65
	Unknown	10	4.14
	NILM	4	1.65
HPV status	Negative	59	24.38
	Positive	163	67.36
	Unknown	20	8.27
HPV sub‐types	High risk	103	42.56
	Low risk	0	0
	Negative	4	1.65
	Unknown	135	55.79
Histopathological grading (biopsy or conization) before hysterectomy	Negative	0	0
	CIN 1	15	6.21
	CIN 1–2	3	1.24
	CIN 2	55	22.72
	CIN 2–3	22	9.09
	CIN 3	141	58.26
	CIN – NOS	2	0.83
	Unknown	4	1.65
Number of conization	0	0	0
	1	184	76.03
	2	43	17.77
	3	13	5.37
	4	2	0.83
First conization technique	LEEP	217	89.67
	LASER	19	7.85
	CKC	6	2.48
Second conization technique	LEEP	45	77.59
	LASER	10	17.24
	CKC	2	3.45
	Unknown	1	1.72
Third excision technique	LEEP	12	80
	LASER	1	6.67
	Unknown	2	13.33
Histopathological examination first conization	Negative	1	0.41
	CIN 1	18	7.44
	CIN 1–2	2	0.83
	CIN 2	40	16.53
	CIN 2–3	17	7.02
	CIN 3	160	66.12
	Unknown	4	1.65
Histopathological examination second conization	Negative	2	3.44
	CIN 1	4	6.90
	CIN 1–2	1	1.72
	CIN 2	12	20.69
	CIN 2–3	3	5.17
	CIN 3	35	60.36
	Unknown	1	1.72
Histopathological examination third conization	Negative	1	6.67
	CIN 1	3	20
	CIN 2	3	20
	CIN 3	7	46.66
	CIN – NOS	1	6.67

Table 4 shows the surgical characteristics of the study group. Hysterectomy was performed with a minimally invasive surgical approach in 62% of patients. An additional indication for hysterectomy was reported in 15.7% of patients and fibromatosis/adenomyosis was the most represented (44.7%).

**TABLE 4:** Surgical characteristics of the sample

Characteristics	Variables	Frequency (n°)	Percentage (%)
Year (at surgery)	2010	6	2.48
	2011	23	9.50
	2012	14	5.78
	2013	19	7.85
	2014	22	9.09
	2015	25	10.33
	2016	26	10.75
	2017	22	9.09
	2018	27	11.16
	2019	26	10.75
	2020	32	13.22
Surgical technique	Laparoscopic	142	58.68
	Robotic	3	1.24
	Laparoscopic‐assisted vaginal hysterectomy	5	2.07
	Laparotomy	77	31.81
	Vaginal hysterectomy	15	6.20
Intraoperative complications	No	239	98.76
	Yes	3	1.24
Blood transfusion	No	239	98.76
	Yes	3	1.24
Postoperative complications	No	232	95.87
	Yes	10	4.13
Operative time (min.) mean (±SD)/median (range)		112.15 ± 44.45/100 (45–260)	

Surgical morbidity was reported in 13 patients (5.4%). Three patients (1.2%) had intraoperative complications/injuries: ureteral and bladder injury (one case), cardiac arrhythmia (one case), and vascular injury (one case). Postoperative complications were recorded in 10 patients (4.1%): three cases of infection resolved after antibiotic therapy, two cases of severe anemia that required blood transfusions, one case of cerebral hemorrhage, one case of deep vein thrombosis, one case of incisional hernia that required reoperation, one case of onset of Takotsubo cardiomyopathy. One patient died, with a 0.4% mortality rate. She was 70 years old and died for cardiocirculatory arrest after hemoperitoneum, despite blood transfusions; she had risk factors for cardiovascular disease (advanced age, blood hypertension, smoke habit) and underwent previous abdominal surgery.

Histopathological findings on hysterectomies are reported in Table 5. A CIN of any grade was recorded in 71.5% of cases. A concomitant vaginal intraepithelial neoplasia (VaIN) was detected in three cases (1.2%): one patient with high‐grade (HG)‐VaIN without persistent CIN, one with HG‐VaIN associated with CIN3, and one with low grade (LG)‐VaIN with CIN1.

**TABLE 5:** Post‐operative characteristics of the sample

Characteristics	Variables	Frequency (n°)	Percentage (%)
Post‐operative histopathological examination	Negative	27	11.17
	Negative + VaIN2	1	0.41
	CIN 1	38	15.70
	CIN 1 ‐ VaIN1	1	0.41
	CIN 1/2	9	3.71
	CIN 2	27	11.17
	CIN 2/3	16	6.61
	CIN 2–3/ adenocarcinoma	1	0.41
	CIN 3	78	32.24
	CIN 3 ‐ VaIN 3	3	1.23
	CIN 1 + sentinel lymph node micro metastasis	1	0.41
	Occult microinvasive carcinoma	39	16.12
	Occult invasive carcinoma	1	0.41
Histopathological upgrading	No	184	76.03
	Yes	58	23.97
Histopathological downgrading	No	174	71.90
	Yes	68	28.10
Occult uterine carcinoma	No	238	98.35
	Yes	4	1.65
Median follow‐up (range)	36 (6–132)		
Vaginal lesion at follow‐up	No	230	95.04
	LG/VaIN/1	5	2.07
	HG/VaIN	5	2.07
	Cancer	2	0.82

No residual disease (negative or CIN1) was found on definitive histopathology examination in 26.8% of cases, suggesting over treatment. A histopathological upgrading was observed in 24% of cases. An occult micro‐invasive or invasive cervical disease was observed in 17.3% of cases, with three invasive lesions (1.24%). In the other four cases (1.6%), incidental occult neoplasia (corpus and adnexa) was found (one case of endometrium adenocarcinoma, two cases of atypical endometrial intraepithelial neoplasia, and one case of tubal intraepithelial neoplasia). Internal analysis (bivariate and multivariate analysis) comparing women with and without a final diagnosis of occult micro‐invasive or invasive carcinoma showed no significant associated independent variable (data not shown).

The median follow‐up was 36 months (range: 6–132 months) and was available for 217/242 patients (89.7%). During follow‐up, a vaginal lesion was recorded in 5% of cases: two invasive vaginal cancer (0.8%), five LG‐VaIN (2.07%), and five HG‐VaIN (2.07%). The first case of invasive vaginal cancer was a G2 squamous carcinoma with parametrial infiltration, which was diagnosed after colpectomy performed for VaIN 3 biopsy, after 23 months of follow‐up. After 48 months from colpectomy, a further recurrence of VaIN 3 was diagnosed. In the second case, an invasive squamous cell carcinoma in the vaginal vault was detected after 14 months of follow‐up, and radiotherapy was performed. On hysterectomy, patients with vaginal cancer during follow‐up had no histopathological findings and a diagnosis of micro‐invasive carcinoma, respectively.

## 4 DISCUSSION

The present study was aimed at observing the actual clinical management, the outcomes, and the trend in hysterectomy rates of patients who underwent hysterectomies for CIN over the last decade across nine Italian referral centers. The charts of 242 women having a hysterectomy for CIN in the last 10 years were reviewed, thus observing several noteworthy findings. First, a significant increase in the trend of HR for CIN was recorded. Second, between 2010 and 2020, about the 2% of hysterectomies was performed for CIN, and about 85% of these patients underwent surgery with no additional indication. Third, the mortality rate was 0.4%, and 5% had operative complications. Fourth, on definitive histopathology examination, a CIN of any grade was recorded in 71.5% of cases and an occult invasive cancer in 1.24%. Overtreatment (no pathology or CIN1) was recorded and was found in 26.8% of cases, also considering that CIN1 does not represent a target for cervical cancer screening because of the low rate of progression and the high rate of spontaneous regression (14). During follow‐up, a vaginal lesion was recorded in 5% of cases. These rates are not negligible and gain particular significance considering the incidence of complications and the mortality rate related to hysterectomy. Likewise, the incidence of vaginal disease in follow‐up was significant, and it is known that both diagnosis and treatment of VaIN and invasive vaginal cancer are more difficult in hysterectomized women.

According to the SICPCV recommendations,^14^ hysterectomy is defined as an unacceptable treatment for HSIL (CIN2‐3) except for a few limited cases where a conservative approach seems to be not possible to perform. The treatment of choice for cervical preneoplastic lesions should be conization or excision, placing increasing attention on limiting too‐aggressive treatment. Similarly, in the last decade, a more conservative surgery has been introduced to treat early‐stage cervical cancer.^23^ The most recent European Guidelines suggest the diagnosis of micro‐invasive carcinoma should be conization or excision, preferring loop, or laser technique, eventually associated with lymph node staging, while radical hysterectomy is identified as an overtreatment.^24^

Despite these assumptions, in our series, a significantly increase in the trend of hysterectomy rate for CIN was recorded and varied from 0.97% in the year 2010 to 3.32% in 2020. This unexpected trend is surprising because in less than 4% of cases, a conservative approach was declared “technically not feasible”. Our results seem to show that even today resorting to hysterectomy is not a rare occurrence, and often this indication seems to occur outside the recommendations.^14^

Although it is acknowledged that in all cases of pre‐invasive cervical neoplasia hysterectomy is not considered an acceptable treatment,^8^, ^9^, ^10^, ^11^, ^12^, ^13^ we evaluated the outcomes of this conduct. In our series, about 5% of patients had complications, ranging from mild to severe. A study conducted in Denmark and published in 2020^25^ on 51 141 patients who underwent hysterectomy for benign disease observed major complications occurred in 3577 (7.0%) of patients and minor complications occurred in 4788 (9.4%). A lower incidence of intra and post‐operative complications emerged from our data, probably because patients were treated in referral centers. However, even one case of death was recorded. Therefore, it is always necessary to personalize the surgical treatment and evaluate the risk for each patient.

In about 17% of histological samples, the final histopathological result revealed a micro‐invasive or invasive occult cancer. No significant independent variables associated with micro‐invasive or invasive cervical lesions were found, probably because of the small sample size. These data are slightly higher than the 12% reported in the literature.^4^ One of the most recent studies^26^ found an incidence of micro‐invasive carcinoma of 10.38% at the final histopathological analysis of hysterectomies performed for CIN 3. The finding of unexpected micro‐invasive or invasive cervical cancer in hysterectomy specimens could explain the reticence of some gynecologists to abandon hysterectomy for treating CIN, especially for women in post‐menopausal status or in those who do not desire further reproduction. Not by chance, in the last 2 years, most hysterectomies for CIN were practiced preferentially in menopausal women. This higher percentage could be partially explained by the COVID‐19 pandemic that radically changed global healthcare, influencing also screening and colposcopy programs.^27^, ^28^ Suspension or postponement of all non‐urgent diagnostic procedures and interventions could have led gynecologists, but also women, to choose hysterectomy as treatment for CIN, considering outpatient management not be easily workable.

Moreover, it may be worthy of note that since 2018, the HR‐ HPV‐DNA test has replaced the Pap smear as the routine test in screening programs in many Italian regions, starting from the 50–64 aged cohort.^29^ A more aggressive surgical approach, especially in postmenopausal women with previous conization and a major risk of recurrence, may be partially explained also by the uncertainties in the management of the results of a new test, leading to an over‐perceived threat by a positive result.

In our series, in about 1% of cases, an occult uterine cancer (corpus and adnexa) was found. In the United States of America, every year over 600 000 benign hysterectomies are performed. Deasi et al. reported in 2019 an incidence of occult endometrial carcinoma in 0.75% of cases, while occult uterine sarcoma was described in 0.22%.^30^ The overall risk of occult uterine cancer was 0.96% in women undergoing hysterectomy for presumed benign indications. It could be noticed retrospective studies report a higher prevalence of occult uterine cancer than prospective studies.^31^, ^32^

In about 5% of cases of this series, a vaginal lesion was recorded during follow‐up. It is established that the risk of vaginal cancer or VaIN is significantly increased in women who have had a hysterectomy for cervical cancer or CIN compared to patients with other indications for hysterectomy.^9^, ^15^, ^16^, ^17^, ^18^, ^19^, ^20^ Hysterectomized women with prevalent CIN have a 20‐fold increase in age‐adjusted risk, compared with hysterectomized women with benign cervical history, and the risk for hysterectomized women with a history of CIN3 is 15‐fold increased, compared to non‐hysterectomized women.^21^ Therefore, in these women, practicing an unjustified hysterectomy could lead to a higher risk of subsequent vaginal cancer. This risk remains elevated for at least 15 years.^21^ From our data, this risk appears lower than expected, probably because follow‐up time is brief in some women. Only 2% of women appeared to have an HG‐VaIN, while less than 1% of cases revealed vaginal cancer, but the short follow‐up does not allow firm conclusions. This could also be because of the particular attention paid to the control of the vaginal walls during excisional treatments, especially in referral centers for colposcopy. Moreover, the Italian recommendations of the SICPCV^14^ pay close attention to VaIN, recommending vaginal biopsy in case of colposcopic alterations; in the presence of multiple vaginal lesions, the biopsy should be targeted on vascular patterns, raised areas, or areas suggestive of vaginal invasive cancer.

The inherent biases of the retrospective study design represent the major weakness of this investigation. Another important limitation was the limited follow up of the study population. However, the relatively large sample size and the originality of the study (very few case series analyzed in the literature) are the main strengths of the research.

Considering the data collected in this Italian multicenter study and the trend recorded, hysterectomy for CIN can lead to complications, risk of the onset of vaginal lesions, and risk of overtreatment, and remains, in the first instance, an unacceptable treatment, to be proposed only after adequate counseling. The treatment of CIN should be as conservative as possible, even after menopause. At the same time, the undeniable increasing trend in using this procedure, even in referral centers, could underline the importance of tailoring treatments, also improving adherence to guideline recommendations. Further studies are needed to evaluate the trend of hysterectomies for CIN in the future, despite data collected in this study strengthening the importance of a conservative approach.

## AUTHOR CONTRIBUTIONS

Conception and design of the work: Ciavattini Andrea, Di Giuseppe Jacopo. Data acquisition: Paolucci Michela, Fichera Maria Sole, De Vincenzo Rosa, Scambia Giovanni, Evangelista Maria Teresa, Bogani Giorgio, Bertolina Francesca, Raspagliesi Francesco, Gardella Barbara, Spinillo Arsenio, Dominoni Mattia, Monti Ermelinda, Liverani Carlo Antonio, Vercellini Paolo, Iorio Maria, Vitobello Domenico, Portuesi Rosalba, Bresciani Gianluigi, Origoni Massimo, Cantatore Francesco, Pellegri Antonio Maurizio, Moriconi Lorenzo, Serri Matteo, Chiari Andrea. Analysis and interpretation of data: Ciavattini Andrea, Di Giuseppe Jacopo, Marconi Chiara, Giannella Luca, Delli Carpini Giovanni, Sopracordevole Francesco, Barbero Maggiorino, Parazzini Fabio. Writing ‐ original draft: Ciavattini Andrea, Di Giuseppe Jacopo, Marconi Chiara. Writing ‐ review & editing: Giannella Luca, Delli Carpini Giovanni, De Vincenzo Rosa, Parazzini Fabio. All authors approved the final version to be published and revised it critically for important intellectual content; all authors agreed to be accountable for all aspects of the work in ensuring that questions related to the accuracy or integrity of any part of the work are appropriately investigated and resolved.

## CONFLICT OF INTEREST

All authors know and comply with the Journal's Conflict of Interest Policy. The present article is not under consideration for publication elsewhere. No conflicts of interest are declared. No sources of financial support are declared.

## Data Availability

Research data are not shared.

## References

[1] Castle PE , Schiffman M , Wheeler CM , Solomon D . Evidence for frequent regression of cervical intraepithelial neoplasia‐grade 2. Obstet Gynecol. 2009;113:18‐25. 19104355 10.1097/AOG.0b013e31818f5008 PMC2694845

[2] McCredie MR , Sharples KJ , Paul C , et al. Natural history of cervical neoplasia and risk of invasive cancer in women with cervical intraepithelial neoplasia 3: a retrospective cohort study. Lancet Oncol. 2008;9:425‐4342. 18407790 10.1016/S1470-2045(08)70103-7

[3] Katki HA , Schiffman M , Castle PE . Five‐year risks of CIN 3+ and cervical cancer among women with HPV testing of ASC‐US pap results. J Low Genit Tract Dis. 2013;17(Supplement 1):S36‐S42. 10.1097/lgt.0b013e3182854253 23519303 PMC3616508

[4] Wright TC , Cox JT , Massad LS , Carlson J , Twiggs LB , Wilkinson EJ . 2001 consensus guidelines for the management of women with cervical intraepithelial neoplasia. Am J Obstet Gynecol. 2003;189:295‐304. 12861176 10.1067/mob.2003.633

[5] Jordan J , Martin‐Hirsch P , Arbyn M , et al. European guidelines for clinical management of abnormal cervical cytology, part 2. Cytopathology. 2009;20:5‐16. 19133067 10.1111/j.1365-2303.2008.00636.x

[6] Sopracordevole F , Clemente N , Delli Carpini G , et al. Trend of decreasing length of cervical cone excision during the last 20years. Eur Rev Med Pharmacol Sci. 2017;21:4747–4754. 29164591

[7] Minardi V , Possenti V , Masocco M , et al. Educational level influences the use of hysterectomy in Italy (data from PASSI survey, 2008–2015). Epidemiol Prev. 2016;40:381. 27764935 10.19191/EP16.5.P381.113

[8] Massad LS , Einstein MH , Huh WK , et al. 2012 updated consensus guidelines for the management of abnormal cervical cancer screening tests and cancer precursors. J Low Genit Tract Dis. 2013;17:S1‐S27. 23519301 10.1097/LGT.0b013e318287d329

[9] Schockaert S , Poppe W , Arbyn M , Verguts T , Verguts J . Incidence of vaginal intraepithelial neoplasia after hysterectomy for cervical intraepithelial neoplasia: a retrospective study. Am J Obstet Gynecol. 2008;199(113):e1‐e5. 10.1016/j.ajog.2008.02.026 18456229

[10] Arbyn M , Kyrgiou M , Gondry J , Petry K , Paraskevaidis E . Long term outcomes for women treated for cervical precancer. BMJ. 2014;348:f7700. 24423750 10.1136/bmj.f7700

[11] Strander B , Hällgren J , Sparén P . Effect of ageing on cervical or vaginal cancer in Swedish women previously treated for cervical intraepithelial neoplasia grade 3: population based cohort study of long term incidence and mortality. BMJ. 2014;348:f7361. 24423603 10.1136/bmj.f7361 PMC3898577

[12] Martin‐Hirsch P , Paraskevaidis E , Bryant A , Dickinson H . Surgery for cervical intraepithelial neoplasia. Cochrane Database Syst Rev. 2013;12:1318. 10.1002/14651858.CD001318.pub3 PMC8958508 24302546

[13] Katki A , Schiffman M , Castle P , et al. Five‐year risk of recurrence after treatment of CIN 2, CIN 3, or AIS: performance of HPV and pap Cotesting in posttreatment management. J Low Genit Tract Dis. 2013;5:17‐S84. 10.1097/LGT.0b013e31828543c5 PMC3616418 23519309

[14] 2019, 2006, and 2002 recommendations for management of the lesions of the lower genital tract disease. Italian Society for Colposcopy and Cervical Vaginal Pathology (SICPCV). Available on https://www.colposcopiaitaliana.com/linee‐guida‐notiziario‐e‐iscrizione (Accessed on 15 January 2022)

[15] Cao D , Wu D , Xu Y . Vaginal intraepithelial neoplasia in patients after total hysterectomy. Curr Probl Cancer. 2021;45:100687. 33309077 10.1016/j.currproblcancer.2020.100687

[16] Kalogirou D , Antoniou G , Karakitsos P , Botsis D , Papadimitriou A , Giannikos L . Vaginal intraepithelial neoplasia (VAIN) following hysterectomy in patients treated for carcinoma in situ of the cervix. Eur J Gynaecol Oncol. 1997;18:188‐191. 9174833

[17] Coronel‐Brizio P , Olivares Nowak J , Palafox SF . Recurrence of high‐grade squamous intraepithelial lesions following hysterectomy. Ginecol Obstet Mex. 1999;67:415‐418. 10544536

[18] Gemmel J , Holmes DM , Duncan ID . How frequently need vaginal smears be taken after hysterectomy for cervical intraepithelial neoplasia? Br J Obstet Gynaecol. 1990;97:58‐61. 2306428 10.1111/j.1471-0528.1990.tb01717.x

[19] Wiener JJ , Sweetnam PM , Jones JM . Long term follow‐up of women after hysterectomy with a history of pre‐invasive cancer of the cervix. Br J Obstet Gynaecol. 1992;99:907‐910. 1450141 10.1111/j.1471-0528.1992.tb14440.x

[20] Babarinsa I , Mathew J , Wilson C , Oladipo A . Outcome of vaginal intraepithelial neoplasia following hysterectomy for cervical intraepithelial neoplasia. J Obstet Gynaecol. 2006;26:157‐158. 16483977 10.1080/01443610500443717

[21] Alfonzo E , Holmberg E , Sparén P , Milsom I , Strander B . Risk of vaginal cancer among hysterectomised women with cervical intraepithelial neoplasia: a population‐based national cohort study. BJOG. 2020;127(4):448‐454. 31769577 10.1111/1471-0528.16028

[22] Aitken CA , Siebers AG , Matthijsse SM , et al. Management and treatment of cervical intraepithelial neoplasia in The Netherlands after referral for colposcopy. Acta Obstet Gynecol Scand. 2019;98:737‐746. 30687935 10.1111/aogs.13547 PMC6593855

[23] Nica A , Marchocki Z , Gien LT , Kupets R , Vicus D , Covens A . Cervical conization and lymph node assessment for early stage low‐risk cervical cancer. Int J Gynecol Cancer. 2021;31:447‐451. 33649012 10.1136/ijgc-2020-001785

[24] Cibula D , Potter R , Planchamp F , et al. The European Society of Gynecological Oncology/European Society for Radiotherapy and Oncology/European Society of Pathology guidelines for the managment of patients with cervical cancer. Radiother Oncol. 2018;127:404‐416. 29728273 10.1016/j.radonc.2018.03.003

[25] Settnes A , Moeller C , Topsoee MF , et al. Complications after benign hysterectomy, according to procedure: a population‐based prospective cohort study from the Danish hysterectomy database, 2004–2015. BJOG. 2020;127:1269‐1279. 32145133 10.1111/1471-0528.16200

[26] Kesic V , Dokic M , Atanackovic J , Milenkovic S , Kalezic I , Vukovic S . Hysterectomy for treatment of CIN. J Low Genit Tract Dis. 2003;7:32‐35. 17051042 10.1097/00128360-200301000-00008

[27] Ciavattini A , Delli Carpini G , Giannella L , et al. European Federation for Colposcopy (EFC) and European Society of Gynaecological Oncology (ESGO) joint considerations about human papillomavirus (HPV) vaccination, screening programs, colposcopy, and surgery during and after the COVID‐19 pandemic. Int J Gynecol Cancer. 2020;30:1097‐1100. 32487685 10.1136/ijgc-2020-001617 PMC7418593

[28] Ciavattini A , Delli Carpini G , Giannella L , et al. Expert consensus from the Italian Society for Colposcopy and Cervico‐Vaginal Pathology (SICPCV) for colposcopy and outpatient surgery of the lower genital tract during the COVID‐19 pandemic. Int J Gynaecol Obstet. 2020;149:269‐272. 32270477 10.1002/ijgo.13158 PMC9087780

[29] Decree of the Acting Commissioner, June 2017, n. U00240. Operational Program 2016‐2018 (DCA 52/2017). Approval of the guideline document for the cervical cancer screening program of the Lazio Region. Organizational model and diagnostic‐therapeutic protocol. https://www.gisci.it/joomdocs/DCA_U00240_HPV.pdf (accessed on 15 January 2022).

[30] Desai VB , Wright JD , Gross CP , et al. Prevalence, characteristics, and risk factors of occult uterine cancer in presumed benign hysterectomy. Am J Obstet Gynecol. 2019;221:39.e1‐39.e14. 10.1016/j.ajog.2019.02.051 PMC7006101 30853364

[31] Hartmann KE , Fonnesbeck C & Surawicz T et al. Management of Uterine Fibroids [Internet]. Agency for Healthcare Research and Quality (US); 2017 Dec. Report No.: 17(18)‐EHC028‐EF. PMID: 30789683. 30789683

[32] Pritts EA , Vanness DJ , Berek JS , et al. The prevalence of occult leiomyosarcoma at surgery for presumed uterine fibroids: a meta‐analysis. Gynecol Surg. 2015;12:165‐177. 26283890 10.1007/s10397-015-0894-4 PMC4532723

